# Eccentricity-based topological indices of QSPR modelling using anti-cancer drugs

**DOI:** 10.3389/fchem.2026.1860531

**Published:** 2026-07-08

**Authors:** R. Vimala, J. Ravi Sankar

**Affiliations:** Department of Mathematics, School of Advanced Sciences, Vellore Institute of Technology, Vellore, Tamil Nadu, India

**Keywords:** eccentricity-based topological indices, linear regression, ridge regression, elastic net regression, LOOCV

## Abstract

In chemical graph theory, topological indices are essential for relating physicochemical properties and molecule structures. This study predicts the properties of 23 anti-cancer drugs by combining 13 eccentricity-based topological indices obtained from hydrogen-depleted molecular graphs with regression approaches. Among these, eccentricity-based topological indices are particularly effective at capturing molecular graphs’ global structural features. Predictive models are created utilizing linear, ridge, and elastic net regression techniques after the obtained indices are related with specific physicochemical properties. The accuracy of the model was assessed using *R* ^2^, RMSE, and leave-one-out cross-validation (LOOCV). The EGA index exhibits the most robust predictive accuracy of the examined indices. This work shows that eccentricity-based topological indices provide reliable and informative indications for QSPR modelling and the effective characterization of molecular structures.

## 1 Introduction

Cancer is a leading cause of disease and death worldwide in the 21st century, posing a serious risk to public health everywhere. It arises when normal cellular regulatory mechanisms malfunction, allowing damaged cells to grow uncontrolled and break down planned cell death, ultimately leading to the spread of cancer (Trichopoulos et al., 1996). Researchers have discovered over 200 types of cancer, which can result from genetic defects, lifestyle choices, and exposure to carcinogens. Some of the numerous symptoms of cancer include unexplained bleeding, irregular tissue development, weight loss, and a persistent cough. Major risk factors include tobacco use, poor eating habits, excessive alcohol use, obesity, and physical inactivity (Dallavalle et al., 2020). Treatment outcomes and survival rates are significantly improved by early tumor diagnosis. Even with significant advancements in therapeutic strategies, finding robust anti-cancer drugs and preventative measures is still crucial to lessening the disease’s worldwide impact (Kour and Ravi Sankar, 2025a; Pandeeswari and Sankar, 2025).

Chemical graph theory analyzes molecules and examines their properties by combining graph theory with chemistry. Topological indices serve as numerical analyzers in these chemical graphs and have a strong correlation with physicochemical properties of molecules (Azam et al., 2025; Pandeeswari and Ravi Sankar, 2025). These indices, which connect molecular structure, are essential to Quantitative Structure-Property Relationship (QSPR). One of the earliest used distance-based topological indices in chemical graph theory is the Wiener index. An important connection between algebraic graphs and topological indices used in QSPR analysis is established by the Wiener index and related characteristics for zero divisor graphs, which offer insightful information on their structural properties (Johnson Rayer et al., 2023; Venkateswarlu et al., 2017). Regression models are used in this work to determine the optimal relationship between physicochemical properties and topological indices. In particular, linear regression, which is a simple yet effective technique for determining fundamental correlations between variables (Kour and Ravi Sankar, 2025b; Tang et al., 2026). By regularizing for multicollinearity, ridge regression improves prediction accuracy and increases its resilience in the presence of highly correlated data. In datasets with several correlated variables, elastic net regression, which combines the regularization concepts of ridge regression, is often beneficial because it improves model stability and facilitates variable selection. Elastic net and ridge regression were used in this work to investigate if regularization enhances predictive consistency in comparison to the traditional linear model, even though the analysis is based on a univariate situation. However, multicollinearity has no effect because every property is described by a single eccentricity-based index, leading to performance that is comparable with linear regression (Huang et al., 2024).

The use of topological indices in chemical graph theory has been carefully examined. Their efficacy in analyzing the physicochemical properties of molecular structures has been shown in a number of studies. Koam et al developed edge-based eccentric topological indices for zero divisor graphs and established important mathematical formulations for eccentric variants of Zagreb and related indices. Their work provided a strong theoretical basis for the application of eccentricity-based indices (Ali et al., 2019). Pandeeswari et al use the linear regression models for the relationship between drugs and their properties, these findings confirm that degcity indices are efficient tools for predicting NSAID properties (Pandeeswari and Sankar, 2025). Gao et al use the indices such as the eccentric-connectivity and Zagreb eccentricity indices, which have been effectively applied to complex networks like dendrimers, focusing on deriving closed-form expressions and corresponding polynomials, which enhance the applicability of such indices in pharmaceutical and nanomaterial analysis (Gao et al., 2019). Likewise, Puneeth et al applied eccentricity-based indices in QSPR analysis of blood cancer drugs, confirming their predictive capability through cubic regression modelling (Puneeth et al., 2024). Xiaolong Shi et al recently considered the latest techniques to QSPR modelling using topological indices for the development of cancer drugs. Their work shows the importance of graph-theoretical indices in the prediction of the physicochemical and pharmacological properties of cancer drugs. The study combined statistical and machine learning techniques and showed that topological indices may have been used to increase the accuracy of prediction and assist in drug design and development (Shi et al., 2025). Huili Li et al have recently worked on the prediction of flavonoid physicochemical properties using degree-based topological indices and regression modelling. Their work showed that QSPR models, especially quadratic regression models, provide excellent predictions for properties like molar refractivity, molar volume, and enthalpy of vaporization (Li et al., 2025). Recently, Guoping Zhang et al analyzed the application of the multiplicative and the classical degree-based topological indices of the chemical graphs in the QSPR modelling of anti-depressant drugs. They showed that the application of machine learning algorithms, especially XGBoost and Random Forest, can greatly enhance the prediction of crucial physicochemical properties such as the boiling point, melting point, critical temperature, critical volume, and molar refractivity. The first Zagreb index was found to be one of the most effective indices with high correlations with numerous pharmacological features (Zhang et al., 2026). Although these studies rely on the effectiveness of topological indices, further comparative evaluation using different regression frameworks remains essential (Huang et al., 2025; Noureen et al., 2026). The present study builds upon these works by examining the predictive performance of selected eccentricity-based indices for anti-cancer drugs within a structured QSPR framework.

In previous work, QSPR models were used for a certain collection of drugs and the physicochemical properties that go along with them using distance-based topological indices (Kour and Sankar, 2024). The systematic review of eccentricity-based topological indices for physicochemical property prediction using regression frameworks has received little attention, despite numerous studies on QSPR modelling of drug molecules. Specifically, there are still few comparison studies using topological indices and regularized regression techniques like ridge and elastic net. Consequently, there is a clear gap in determining the best eccentricity-based index for every physicochemical property and evaluating its prediction ability using various regression techniques. The comprehensive view of several eccentricity-based topological indices across a larger dataset and a comparative analysis utilizing linear, ridge, and elastic net regression models are what make this study novel. The study also gives a strong framework for structure–property prediction in chemical graph theory. It does this by using statistical performance criteria to find the best index for each physicochemical property. The current study extends by using the anti-cancer drugs and physicochemical property information, substituting eccentricity-based topological indices for distance-based indices. The goal is to investigate the structural significance and prediction power of eccentricity-based indices in the context of QSPR modelling. The hydrogen-depleted molecular graphs were obtained by eliminating hydrogen atoms and focusing only on the heavy atoms and their bonding connections. In chemical graph theory, these types of graphs are frequently employed to simplify molecular structures while preserving significant connectivity information for topological analysis. The eccentricity-based topological indices were computed using the molecular structures obtained from the PubChem database.

Outline of the paper: In Section 2, materials and methods, the process of collection of data and analysis of chosen topological indices are discussed. Section 3 highlights the models’ effectiveness and accuracy in predicting important outcomes. Section 4 discussed the LOOCV method. Section 5 discusses the results by evaluating the model’s performance and its implications for predictive analysis. Finally, Section 6 highlights the key findings and discusses the study’s limitations.

## 2 Materials and methods

This study examines 23 anti-cancer drugs that were chosen for their substantial use in oncology, therapeutic significance, and clinical relevance. These molecular structures show atomic arrangements and functional groups for their biological activity. The PubChem database is the source of all drug information, including names, structural formulas, and its applications. Table 1 summarizes the structural features and medical uses of the chosen drugs.

**TABLE 1:** Drugs, molecular formulas, and therapeutic uses.

Drug	Molecular formula	Therapeutic uses
Altretamine	${\text{C}}_{9}{\text{H}}_{18}{\text{N}}_{6}$	Palliative treatment for recurrent or persistent ovarian cancer following cisplatin and alkylating agents
Busulfan	${\text{C}}_{6}{\text{H}}_{14}{\text{O}}_{6}{\text{S}}_{2}$	Used in conditioning regimens before hematopoietic progenitor cell transplantation for chronic myelogenous leukemia
Carmustine	${\text{C}}_{5}{\text{H}}_{9}{\text{Cl}}_{2}{\text{N}}_{3}{\text{O}}_{2}$	Treatment of brain tumors due to its ability to cross the blood–brain barrier
Cyclophosphamide	${\text{C}}_{7}{\text{H}}_{15}{\text{Cl}}_{2}{\text{N}}_{2}{\text{O}}_{2}$ P	Used in combination therapies for various cancers at initial and advanced stages
Dacarbazine	${\text{C}}_{6}{\text{H}}_{10}{\text{N}}_{6}$ O	Primarily used for metastatic malignant melanoma and Hodgkin’s disease
Ifosfamide	${\text{C}}_{7}{\text{H}}_{15}{\text{Cl}}_{2}{\text{N}}_{2}{\text{O}}_{2}$ P	Applied in the treatment of testicular cancer, sarcomas, and lymphomas
Mechlorethamine	${\text{C}}_{5}{\text{H}}_{11}{\text{Cl}}_{2}$ N	Palliative therapy for advanced Hodgkin’s disease and chronic leukemias
Procarbazine	${\text{C}}_{12}{\text{H}}_{19}{\text{N}}_{3}$ O	Utilized in combination regimens to treat advanced Hodgkin’s disease
Temozolomide	${\text{C}}_{6}{\text{H}}_{6}{\text{N}}_{6}{\text{O}}_{2}$	Treatment of glioblastoma and refractory anaplastic astrocytoma
Thiotepa	${\text{C}}_{6}{\text{H}}_{12}{\text{N}}_{3}$ PS	Pre-transplant conditioning for hematopoietic progenitor cell transplantation
Melatonin	${\text{C}}_{13}{\text{H}}_{16}{\text{N}}_{2}{\text{O}}_{2}$	Used in the treatment of insomnia, jet lag, and circadian rhythm sleep disorders; also exhibits antioxidant properties
Podophyllotoxin	${\text{C}}_{22}{\text{H}}_{22}{\text{O}}_{8}$	Treatment of external genital and perianal warts caused by human papillomavirus (HPV)
Prednisone	${\text{C}}_{21}{\text{H}}_{26}{\text{O}}_{5}$	Treatment of leukemia, lymphoma, and other inflammatory or immune-related conditions
Dexamethasone	${\text{C}}_{22}{\text{H}}_{29}{\text{FO}}_{5}$	Anticancer and anti-inflammatory agent used in leukemia, lymphoma, and multiple myeloma
Docetaxel	${\text{C}}_{43}{\text{H}}_{53}{\text{NO}}_{14}$	Used in the treatment of breast, lung, prostate, head and neck, and gastric cancers
Paclitaxel	${\text{C}}_{47}{\text{H}}_{51}{\text{NO}}_{14}$	Used in the treatment of breast, ovarian, lung cancers, and AIDS-related Kaposi sarcoma
Azacytidine	${\text{C}}_{8}{\text{H}}_{12}{\text{N}}_{4}{\text{O}}_{5}$	Treatment of acute myeloid leukemia (AML) and myelodysplastic syndromes (MDS)
Tamoxifen	${\text{C}}_{26}{\text{H}}_{29}$ NO	Antineoplastic agent used for the treatment of hormone receptor-positive breast cancer and for reducing the risk of breast cancer recurrence
Etoposide	${\text{C}}_{29}{\text{H}}_{32}{\text{O}}_{13}$	Chemotherapy for small cell lung cancer and advanced testicular cancer
Actinomycin D	${\text{C}}_{62}{\text{H}}_{86}{\text{N}}_{12}{\text{O}}_{16}$	Treatment of Ewing sarcoma, gestational trophoblastic disease, rhabdomyosarcoma, testicular cancer, and Wilms tumor
Simvastatin	${\text{C}}_{25}{\text{H}}_{38}{\text{O}}_{5}$	Management of elevated cholesterol levels and cardiovascular disease prevention
Nitroxoline	${\text{C}}_{9}{\text{H}}_{6}{\text{N}}_{2}{\text{O}}_{3}$	Treatment of bacterial infections; also exhibits antitumor activity through inhibition of angiogenesis
Deguelin	${\text{C}}_{23}{\text{H}}_{22}{\text{O}}_{6}$	Investigational anticancer agent with anti-angiogenic, antiviral, and anti-inflammatory activities

**FIGURE 1:**
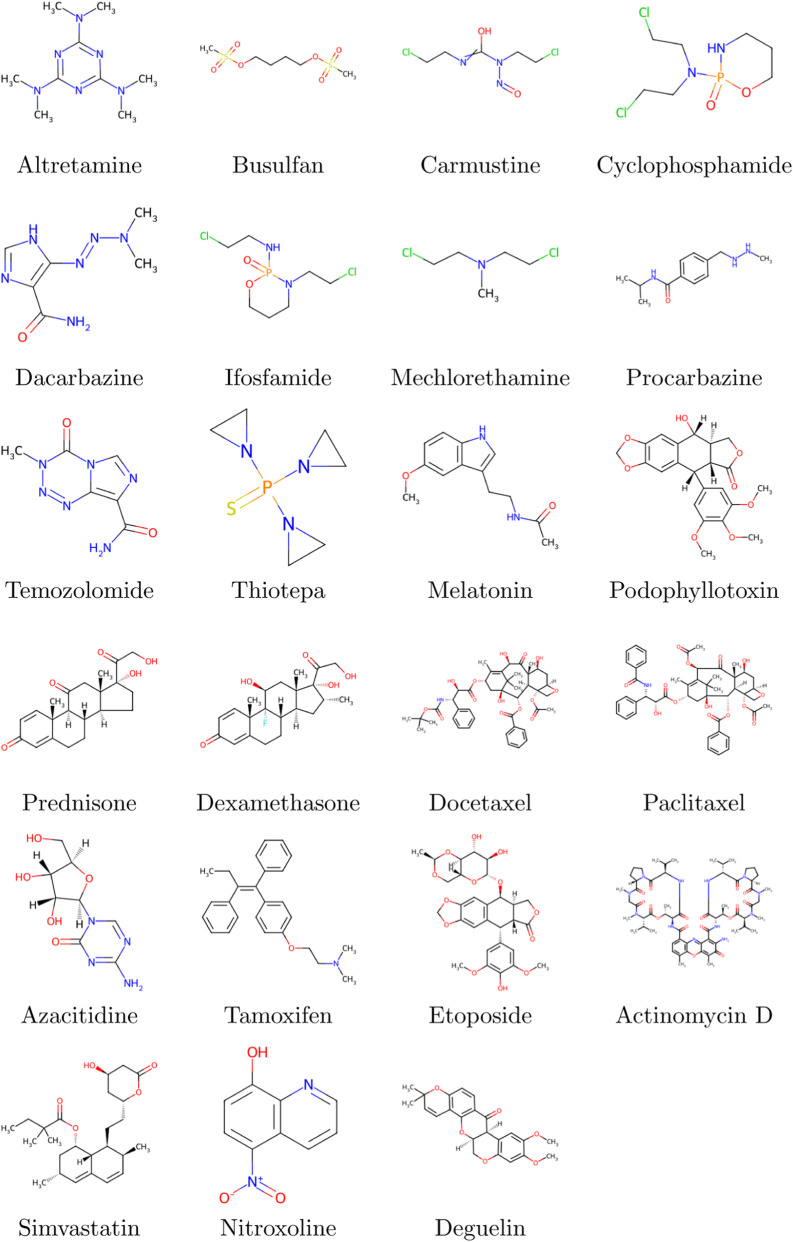
Molecular graphs of anti-cancer drugs.

QSPR study was supported by a set of basic physicochemical properties. Table 2 shows the physicochemical properties of the selected drugs, which is taken from Kour and Sankar (2024). In this table, BP represents boiling point, MP represents melting point, E represents enthalpy, FP denotes flash point, MR represents molar refractivity, P represents polarizability, ST represents surface tension, and MV represents molar volume. It is well recognized that these properties have a substantial impact on drug performance and molecular behavior. In order to highlight the fundamental relation of the molecular framework, hydrogen atoms were removed from the molecular graphs corresponding to each drug. These graphs serve as the basis for calculating topological indices, as seen in Figure 1. The resulting structural representation makes it possible to assess molecular topology and enables accurate modelling.

**TABLE 2:** Anti-cancer drugs and their physicochemical properties.

Drug	BP	MP	E	FP	MR	P	ST	MV
Altretamine	339.4	168	58.3	159.1	63.5	25.2	53.8	183.1
Busulfan	464.0	118	69.8	234.4	50.9	20.2	46.6	182.4
Carmustine	309.6	30	63.8	141.0	46.6	18.5	50.4	146.4
Cyclophosphamide	336.1	51	57.9	157.1	58.1	23.0	44.3	195.7
Dacarbazine	456.3	205	71.6	229.7	46.2	18.3	60.7	122.6
Ifosfamide	336.1	40	57.9	157.1	58.1	23.0	44.3	195.7
Mechlorethamine	110.3	108	34.9	20.5	38.6	15.3	31.3	141.1
Procarbazine	384.6	223	63.3	148.9	65.8	26.1	37.9	213.6
Temozolomide	526.6	212	80.1	272.3	45.6	18.1	105.1	98.4
Thiotepa	270.2	51.5	50.8	117.2	49.1	19.5	77.8	125.8
Melatonin	512.8	117.25	78.4	264.0	67.6	26.8	46.7	197.6
Podophyllotoxin	597.9	183.5	93.6	210.2	104.3	41.3	52.8	302.4
Prednisone	573.7	237.0	98.7	314.8	94.1	37.3	58.6	273.6
Dexamethasone	568.2	263.0	98.0	297.5	100.2	39.7	56.6	296.2
Docetaxel	900.5	189.0	137.1	498.4	205.2	81.4	66.2	585.7
Paclitaxel	957.1	214.5	146.0	532.6	219.3	86.9	68.5	610.6
Azacitidine	534.5	229.0	93.2	277.0	51.1	20.3	106.8	117.1
Tamoxifen	482.3	97.5	74.7	140.0	118.9	47.1	40.4	356.2
Etoposide	798.1	243.5	121.7	263.6	140.1	55.5	76.5	378.5
Actinomycin D	1386.0	252.0	211.5	792.1	323.7	128.3	53.9	880.7
Simvastatin	564.9	129.5	97.5	184.8	116.4	46.1	43.1	376.6
Nitroxoline	419.0	182.0	69.9	207.2	50.6	20.1	76.5	127.0
Deguelin	560.1	86.0	84.3	244.7	105.1	41.7	43.2	314.2

### 2.1 Eccentricity-based topological indices

The 13 eccentricity-based topological indices listed in Table 3 are used to examine the molecular structures of the selected anti-cancer drugs. These indices give detailed information on molecular size, connectivity, and branching that is significant for structure–property relationships by characterizing global structural properties and vertex distance of molecular graphs. The fundamental framework regulating physicochemical properties is thought to be the focus of hydrogen-depleted molecular graphs (Dankelmann et al., 2004; Gao et al., 2018; Zheng et al., 2019). Table 4 summarizes the computed values of these indices. Python was used for all computational and statistical analyses of the chosen indices. Linear regression, ridge regression, and elastic net regression were found using standard libraries numpy, pandas, sklearn, and RDKit. All computations were performed using Python 3.10 with scikit-learn 1.3.0. Ridge regression was implemented with α=1.0 , and elastic net with α=1.0 and $l_{1}\text{\_}\text{ratio}=0.5$ . Heatmaps and other graphical representations were utilized for correlation analysis of the topological indices and physicochemical properties under consideration, while computational techniques were used for statistical evaluation.

**TABLE 3:** Eccentricity-based topological indices.

Topological indices	Formula
Eccentric connectivity index	$ECI=\sum_{v\in V\left(G\right)}d\left(v\right)\;e\left(v\right)$
Augmented eccentric connectivity index	$AECI=\sum_{u\in V\left(G\right)}\frac{M\left(u\right)}{e\left(u\right)}$
Total eccentricity index	$TEI=\sum_{v\in V\left(G\right)}e\left(v\right)$
Inverse eccentricity index	$IEI=\sum_{v\in V\left(G\right)}\frac{1}{e\left(v\right)}$
Average eccentricity index	$AEI=\frac{1}{n}\sum_{v\in V\left(G\right)}e\left(v\right)$
Eccentric distance sum index	$EDS=\sum_{v\in V\left(G\right)}e\left(v\right)\;D\left(v\right)$
Connective eccentricity index	$CEI=\sum_{v\in V\left(G\right)}\frac{d\left(v\right)}{e\left(v\right)}$
Eccentric-based harmonic index	$EHI=\sum_{uv\in E\left(G\right)}\frac{2}{e\left(u\right)+e\left(v\right)}$
First Zagreb eccentricity index	$EM_{1}=\sum_{v\in V\left(G\right)}{\left(e\left(v\right)\right)}^{2}$
Second Zagreb eccentricity index	$EM_{2}=\sum_{uv\in E\left(G\right)}e\left(u\right)\;e\left(v\right)$
Geometric Arithmetic eccentricity index	$EGA=\sum_{uv\in E\left(G\right)}\frac{2\sqrt{e\left(u\right)\;e\left(v\right)}}{e\left(u\right)+e\left(v\right)}$
ABC eccentricity index	$EABC=\sum_{uv\in E\left(G\right)}\sqrt{\frac{e\left(u\right)+e\left(v\right)-2}{e\left(u\right)\;e\left(v\right)}}$
Modified eccentric connectivity index	$MECI=\sum_{v\in V\left(G\right)}\delta_{v}\;e\left(v\right)$

where, *V* (*G*) → vertex set of graph *G*,

*E* (*G*) → edge set of graph *G*,

*n* → number of vertices in the graph,

*e* (*v*) → eccentricity of the vertex *v*,

*d* (*v*) → degree of the vertex *v*,

*M* (*u*) → product of degrees of neighboring vertices of *u*,

*D* (*v*) → sum of distances from *v* to all other vertices,

*δ* (*v*) → sum of degrees of neighboring vertices of *v*.

**TABLE 4:** Computed eccentricity-based topological indices of anti-cancer drugs.

Drugs	ECI	TEI	IEI	AECI	AEI	MECI	CEI	EHI	$EM_{1}$	$EM_{2}$	EDS	EGA	EABC
Altretamine	147	78	2.9500	19.2000	5.2000	357	6.2500	3.0909	414	360	3780	14.9194	8.6108
Busulfan	190	106	1.9357	8.3857	7.5714	468	3.7048	1.8439	834	713	6154	12.9710	6.3869
Carmustine	129	74	2.0286	9.2238	6.1667	265	3.9143	1.9403	474	388	2904	10.9557	5.9169
Cyclophosphamide	149	78	2.5952	16.5429	5.5714	334	5.4548	2.7038	448	404	3478	13.9406	7.8084
Dacarbazine	143	75	2.3381	14.7143	5.7692	330	4.8881	2.4260	447	400	3128	12.9530	7.1522
Ifosfamide	162	85	2.3952	15.1762	6.0714	362	5.0405	2.4982	535	480	3883	13.9424	7.5680
Mechlorethamine	61	37	1.8167	6.5500	4.6250	121	3.3833	1.6652	179	136	725	6.9486	4.2152
Procarbazine	258	134	2.0084	11.2622	8.3750	574	4.1438	2.0646	1176	1078	8964	15.9723	7.5501
Temozolomide	153	75	2.7286	23.0857	5.3571	376	6.0952	3.0277	419	399	3078	14.9498	8.4954
Thiotepa	86	38	3.3333	39.8333	3.4545	224	8.3333	4.0643	136	144	992	12.8576	8.5385
Melatonin	269	132	2.2798	16.3508	7.7647	622	5.0052	2.4901	1064	1033	9070	17.958	8.7601
Podophyllotoxin	577	262	3.5275	36.4326	8.7333	1427	8.2303	4.1007	2344	2494	38048	33.9426	15.6259
Prednisone	496	230	3.0537	33.4583	8.8462	1308	7.028	3.5003	2110	2181	26154	28.947	13.3177
Dexamethasone	530	248	3.2787	38.0500	8.8571	1423	7.507	3.7389	2274	2327	30646	30.9434	14.2342
Docetaxel	1759	827	4.1855	40.8710	14.2586	4519	9.2893	4.6378	12097	12570	369891	62.956	23.1866
Paclitaxel	2007	930	4.2362	40.8745	15.0000	5060	9.4495	4.7188	14260	15116	454580	67.9592	24.3654
Azacitidine	240	118	2.5210	20.0056	6.9412	582	5.5532	2.7618	842	814	7110	17.9533	9.1622
Tamoxifen	644	307	2.6595	19.1459	10.9643	1464	5.8078	2.8963	3485	3560	48709	29.9637	12.474
Etoposide	1152	517	3.5189	35.1991	12.3095	2877	8.2415	4.1128	6553	7088	138837	47.9564	18.9377
Actinomycin D	4148	1976	4.2271	34.8505	21.9556	10336	9.1758	4.585	44580	46060	2045524	95.9721	28.8312
Simvastatin	632	306	3.0424	23.9635	10.2000	1556	6.7036	3.3429	3222	3208	51506	31.959	13.7923
Nitroxoline	141	69	2.9500	24.8667	4.9286	336	6.6333	3.2773	351	336	2782	14.9176	8.7645
Deguelin	671	303	2.8972	28.5770	10.4483	1699	6.7551	3.3677	3297	3526	45868	32.9554	14.0662

### 2.2 Regression models

The correlation between the chosen topological indices and the physicochemical properties of the anti-cancer drugs was examined using linear regression. Since the topological indices under consideration may show multicollinearity, which might impact the stability and predictive performance of standard linear regression models, ridge regression and elastic net regression were used. While elastic net regression successfully manages correlated indices by combining regularization and variable selection, ridge regression uses regularization to lessen coefficient instability.

• Linear regression

A basic statistical method for predicting a quantitative response variable Y based on a single predictor X is simple linear regression. The two variables are assumed to have a nearly linear relation, which can be expressed as

$$Y\approx \gamma_{0}+\gamma_{1}X$$

where X is the explanatory variable, Y is the response variable and $\gamma_{0}$ is the intercept term and $\gamma_{1}$ is the regression coefficient which indicates how a unit change in X corresponds to a change in Y (Arockiaraj et al., 2023; Narayana et al., 2018).

• Ridge regression

A regularization method called ridge regression was created to manage multicollinearity among predictors and stabilize coefficient estimate in situations when there are many explanatory factors. Ridge regression, in contrast to standard least squares, adds an $\ell_{2}$ -norm penalty, which lowers variance without removing predictors by limiting the size of regression coefficients. This strategy works especially well when predictors show high correlations and have little but significant effects on the response variable. A non-negative regularization parameter, usually chosen using cross-validation, regulates the amount of shrinkage. Instead of doing variable selection, ridge regression keeps all predictors in the model because it does not decrease coefficients to zero.

$${\hat{\gamma }}^{\text{ ridge}}=\arg\underset{\gamma }{\min}\left\{\|y-X\gamma {\|}_{2}^{2}+\lambda \|\gamma {\|}_{2}^{2}\right\}$$

where X is the predictor matrix, y is the response vector and γ is the coefficient vector,

$$\|y-X\gamma {\|}_{2}^{2}=\overset{n}{\underset{i=1}{\sum }}{\left(y_{i}-x_{i}^{⊤}\gamma \right)}^{2}$$

indicates the $\ell_{2}$ -norm loss function (residual sum of squares), $x_{i}^{⊤}$ symbolizes the i -th row of the design matrix X , and

$$\|\gamma {\|}_{2}^{2}=\overset{p}{\underset{j=1}{\sum }}\gamma_{j}^{2}$$

is the $\ell_{2}$ -norm penalty applied to the regression coefficients. The tuning parameter λ≥0 establishes the degree of coefficient shrinkage and regulates the penalty’s intensity. Stronger shrinking occurs when λ is larger. Since the data determines the ideal value of λ , data-driven techniques like cross-validation are frequently used to choose it. It is believed that the intercept term is zero since the response variable was mean-centered (Ogutu et al., 2012).

• Elastic net regression

Elastic net regression is a method to regularize datasets where predictor variables may be very similar to each other. It works especially well with high-dimensional data and helps reduce the effects of multicollinearity between predictors. Elastic net improves model stability and keeps the ability to do good variable selection by combining the $\ell_{1}$ and $\ell_{2}$ regularization penalties (Ogutu et al., 2012).

$${\hat{\beta }}^{\left(\text{enet}\right)}=\left(1+\frac{\lambda_{2}}{n}\right)\arg\underset{\beta }{\min}\left\{\|y-X\beta {\|}_{2}^{2}+\lambda_{2}\|\beta {\|}_{2}^{2}+\lambda_{1}\|\beta {\|}_{1}\right\}$$

where y is the response vector, X is the predictor matrix, β is the regression coefficient vector, $\lambda_{1}$ and $\lambda_{2}$ are non-negative regularization parameters controlling the $\ell_{1}$ - and $\ell_{2}$ -penalties, respectively, and n denotes the number of observations. The term $\|y-X\beta {\|}_{2}^{2}$ represents the residual sum of squares, while $\|\beta {\|}_{1}$ and $\|\beta {\|}_{2}^{2}$ denote the $\ell_{1}$ - and squared $\ell_{2}$ -norms of the coefficient vector, respectively.

## 3 Main results

### 3.1 Correlation analysis

**FIGURE 2:**
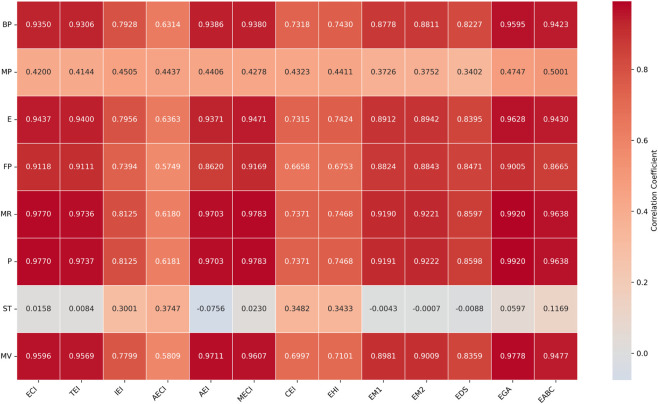
Correlation heatmap of topological indices and physicochemical properties.

To examine the relationship between the chosen topological indices and the physicochemical properties, Pearson’s coefficient was used to do a correlation analysis. The data is shown as a heatmap in Figure 2, which shows the correlations’ direction and strength in a graph. The gradient of colors makes it easy to understand the difference between strong correlations (|r|≥0.7) , moderate correlations (0.5≤|r|<0.7) and weak correlation (|r|<0.5) . This shows that the molecules’ structural behavior might be accurately shown by these indices. This heatmap shows how molecules are related to each other.

From the heatmap, BP, E, MR, P, and MV all show strong positive correlations with maximum number of indices, suggesting that these indices accurately capture structural properties connected with molecular size, branching, electron distribution, and intermolecular interactions. The EGA index has the highest correlations with BP (r = 0.9595), E (r = 0.9628), MV (r = 0.9778), and MR (r = 0.9920) and P (r = 0.9920). These significant correlations indicate that EGA accurately depicts molecular structure related to electron cloud dispersion, atomic connectivity, and general molecular configuration. In addition, ECI, TEI, MECI, and EABC show significant positive correlations with a number of physicochemical properties, showing their potential as dependable molecular identifiers in QSPR modelling. Whereas, MP show moderate and ST show weak correlation among the selected indices.

### 3.2 Performance comparison

The quantitative relation between the calculated eccentricity-based topological indices and the chosen physicochemical properties of the drugs within the QSPR framework was established using regression analysis. Using variables like the coefficient of determination (*R* ^2^), p-value, and the models’ statistical significance, predictive power was assessed. The best eccentricity-based indices for forecasting the attributes under consideration may be found by comparing these regression results. In the following tables, x is the value of the chosen eccentricity-based topological indices, and Y represents the corresponding physicochemical properties of the chosen drugs. The regression model establishes a quantitative relationship between the topological indices x and the molecular property Y. From Tables 5–7, all 13 topological indices were evaluated using regression analysis for each physicochemical property. Each index’s performance was evaluated using statistical measures including RMSE and *R* ^2^. The index with the lowest RMSE and the highest *R* ^2^ value was chosen as the best index for that particular property. This selection shows that the selected index has the most precise and reliable correlation between the appropriate physicochemical properties and molecular structure. Ridge and elastic net regression models were used in addition to linear regression to assess the generated models’ prediction stability and robustness under regularized frameworks.

**TABLE 5:** Linear regression analysis between physicochemical properties and topological indices.

Property	Index	Regression equation	$R^{2}$	F-test	p-value	RMSE
Boiling point	EGA	Y=209.8966+11.7257x	0.9207	243.8234	p<0.01	73.8602
Melting point	EABC	Y=88.0151+5.7816x	0.2510	7.00039	p<0.01	63.0169
Enthalpy	EGA	Y=40.6971+1.6702x	0.9269	266.6427	p<0.01	10.0606
Flash point	MECI	Y=152.5895+0.0643x	0.8406	110.7616	p<0.01	63.4684
Molar refractivity	EGA	Y=8.2127+3.1486x	0.9841	1298.1738	p<0.01	8.5953
Polarizability	EGA	Y=3.2667+1.2478x	0.9841	1300.6143	p<0.01	3.4031
Surface tension	AECI	Y=42.5548+0.6526x	0.1404	3.4299	0.0781	17.8033
Molar volume	EGA	Y=40.4899+08.5142x	0.9561	457.7715	p<0.01	39.1408

**TABLE 6:** Ridge regression analysis between physicochemical properties and topological indices.

Property	Index	Regression equation	$R^{2}$	F-test	p-value	RMSE
Boiling point	EGA	Y=209.9276+11.7246x	0.9207	243.8234	p<0.01	73.8602
Melting point	EABC	Y=88.09161+5.7753x	0.2510	7.00039	p<0.01	63.0169
Enthalpy	EGA	Y=40.7015+1.6701x	0.9269	266.6427	p<0.01	10.0606
Flash point	MECI	Y=15.5895+0.0643x	0.8406	110.7616	p<0.01	63.4684
Molar refractivity	EGA	Y=8.2209+3.1486x	0.9841	1298.1738	p<0.01	8.5953
Polarizability	EGA	Y=3.2700+1.2477x	0.9841	1300.6143	p<0.01	3.4031
Surface tension	AECI	Y=42.5604+0.6523x	0.0140	3.4299	0.0781	17.8033
Molar volume	EGA	Y=40.5124+08.5134x	0.9561	457.7715	p<0.01	39.1408

**TABLE 7:** Elastic net regression analysis between physicochemical properties and topological indices.

Property	Index	Regression equation	$R^{2}$	F-test	p-value	RMSE
Boiling point	EGA	Y=209.8966+11.7257x	0.9207	243.2460	p<0.01	73.8602
Melting point	EABC	Y=88.0151+5.7816x	0.2510	6.8002	p<0.01	63.0169
Enthalpy	EGA	Y=40.6971+1.6702x	0.9269	265.7090	p<0.01	10.0606
Flash point	MECI	Y=152.5895+0.0643x	0.8406	110.7613	p<0.01	63.4684
Molar refractivity	EGA	Y=8.2127+3.1486x	0.9841	1294.3043	p<0.01	8.5953
Polarizability	EGA	Y=3.2667+1.2478x	0.9841	1295.2329	p<0.01	3.4031
Surface tension	AECI	Y=42.5548+0.6526x	0.1404	3.3591	0.0811	17.8033
Molar volume	EGA	Y=40.4899+08.5142x	0.9561	456.6484	p<0.01	39.1408

Using topological indices, the effectiveness of linear, ridge, and elastic net regression models for the prediction of physicochemical properties was assessed. All three models exhibited quite similar values. Overall, this suggests that the predictions are consistent and reliable throughout the dataset. The evaluation showed that the EGA index had the best predictive power for most of the properties, with strong ( $R^{2}$ = 0.9207–0.9841) and strong statistical significance (p<0.01) . This comparison establishes the framework for determining which models and indices perform the best, laying the basis for a thorough analysis of the particular outcomes. The EGA index exhibited the highest correlations with P ( $R^{2}$ = 0.9841) and MR ( $R^{2}$ = 0.9841), showing its high effectiveness in forecasting these optical and electrical properties. The EGA showed strong connections with BP ( $R^{2}$ = 0.9207), E ( $R^{2}$ = 0.9269), and MV ( $R^{2}$ = 0.9561), shows its adaptability and wide range of applications across several thermodynamic properties. Additionally, the MECI index showed a high and significant relation with FP $\left(R^{2}=0.8406\right)$ . Also, the EABC and AECI indices showed significantly weaker relation with ST $\left(R^{2}=0.1404\right)$ and MP $\left(R^{2}=0.2510\right)$ . Using the EGA index, all three models obtained the (RMSE = 8.5953) and $R^{2}$ values (0.9841) for MR. This convergence suggests that the multicollinearity challenges that regularized models are intended to address were not present because each topological index was assessed separately in the study. As a result, minimal penalization was performed by the ridge and elastic net models, which represented the Ordinary Least Squares (OLS) solution and verified the strong stability and robustness of the baseline linear estimates for these particular relationships.

## 4 Leave-one-out cross-validation (LOOCV) methods

LOOCV was used to assess the models’ prediction reliability for the ideal kernel function and its associated properties. In this iterative process, the model is trained using the remaining [ n-1 ] samples after a single sample is methodically removed from the dataset to act as the validation set. The target value of the sample that was withheld is then predicted using the trained model. This procedure is performed until each sample in the dataset has had its value predicted and eliminated exactly once. This thorough validation process optimizes the use of the available data while giving a reliable evaluation of the correctness and generalizability of the model. A statistical metric used to assess the predictive power of a regression model during LOOCV is the cross-validated coefficient of determination $\left(Q^{2}\right)$ . The average prediction error observed during the LOOCV procedure is measured by the LOOCV Root Mean Square Error (LOOCV RMSE). It is determined by taking the square root of the average squared variation between the values seen and those predicted throughout all validation steps.

The internal validation metrics, namely LOOCV RMSE and $Q^{2}$ , are shown in Table 8 to evaluate the predictive reliability and generalizability of the generated regression models. The EGA index showed significant prediction accuracy for MR and P, with high $Q^{2}$ values of 0.9777 and low LOOCV RMSE values (10.1735 and 4.0299, respectively). Additionally, strong prediction accuracy remained stable for BP ( $Q^{2}$ = 0.9039), E ( $Q^{2}$ = 0.9072), and MV ( $Q^{2}$ = 0.9486). These particular models are extremely stable, robust, and free of overfitting, as seen by the close alignment between these $Q^{2}$ validation metrics and the matching training $R^{2}$ values. However, the cross-validation metrics for ST ( $Q^{2}$ = 0.0299 using AECI) and MP ( $Q^{2}$ = 0.1464 using EABC) showed poor prediction. Further, the single-variable linear models based on these particular indices are inadequate for capturing the structural variation underlying those specific physicochemical property comes from the models’ incapacity to correctly predict withheld samples. The linear, ridge, and elastic net models produced nearly identical $Q^{2}$ and LOOCV RMSE values across all analyzed properties when the computational approaches were compared. The optimization techniques applied nearly negligible L 1 and L 2 regularization penalties since these univariate (single-descriptor) models lacked multicollinearity, as was seen during the training phase. As a result, during the cross-validation method, the ridge and elastic net models converged toward the ordinary least squares (linear) solution. This convergence confirms that standard linear regression is fully optimized and offers the highest level of prediction stability for these particular one-to-one connections without requiring detailed regularization.

**TABLE 8:** Comparison of $Q^{2}$ and LOOCV RMSE across linear, ridge, and elastic net models.

Property	Index	Linear		Ridge		Elastic net	
​	​	$Q^{2}$	LOOCV RMSE	$Q^{2}$	LOOCV RMSE	$Q^{2}$	LOOCV RMSE
BP	EGA	0.9039	81.3003	0.9039	81.3043	0.9038	81.3498
MP	EABC	0.1464	67.2340	0.1464	67.2314	0.1470	67.2098
E	EGA	0.9072	11.3437	0.9072	11.3445	0.9069	11.3597
FP	MECI	0.8100	69.3010	0.8100	69.3010	0.8100	69.3009
MR	EGA	0.9777	10.1735	0.9777	10.1761	0.9775	10.2125
P	EGA	0.9777	4.0299	0.9777	4.0309	0.9775	4.0516
ST	AECI	0.0299	18.9127	0.0299	18.9126	0.0299	18.9127
MV	EGA	0.9486	42.3634	0.9486	42.3660	0.9485	42.4005

## 5 Discussion

The QSPR models improve effective computational analysis and lower the dependence on substantial experimental validation by modelling physicochemical properties using eccentricity-based topological indices. This makes it easier to find effective anti-cancer drugs, leading more quickly by using effective structure–property relations. The linear, elastic net, and ridge regression models were used in this study to assess prediction performance. Ridge, and elastic net worked similarly, but linear regression showed slightly better results, with slightly higher *R* ^2^ values and lower RMSE for the majority of properties. Since every property is described using a single index and multicollinearity effects are negligible, the small variations across the models suggest that regularization gives little advantage in this univariate framework. Overall, the findings show that the structural significance of the selected eccentricity-based indices has a bigger impact on predicted accuracy than the regression method’s sophistication. The findings exhibit these indices’ value in facilitating effective drug screening and development and validate their applicability for QSPR modelling of anti-cancer drugs. Thus, the correlation between eccentricity-based topological indices and physicochemical properties such as boiling point, polarizability, molar refractivity and enthalpy has been analyzed using molecule size, branching, electron distribution and intermolecular relations. This facilitates understanding the structural importance of the indices used in QSPR modelling.

The harmonic index was shown to be one of the strongest predictive indices for molar refractivity with low RMSE and maximum correlation coefficient values. The study also showed the improvement in predictive performance of curvilinear regression models by reduction in RMSE and rise in correlation values (Kirana et al., 2024). Similarly, a comparative QSPR study for lung cancer medications with degree-based, neighborhood degree-based, and modified reverse degree-based topological indices was carried out. In their study, the redefined First Zagreb index, atom bond connectivity index, geometric-Bi Zagreb index and harmonic index showed good predictive power for some physicochemical properties (Arockiaraj et al., 2025). For this study, the eccentricity-based topological indices have been considered for anti-cancer drugs. The EGA index among the analyzed indices exhibited comparatively greater prediction performance based on the obtained regression statistical analysis. The results show that the eccentricity-based topological indices also have a strong predictive capacity and can be employed as useful molecular descriptors in QSPR modelling, in addition to the generally used degree-based and neighbourhood degree-based indices.

In all three models, the EGA index showed the strong predictive performance for MR and P, with the highest *R* ^2^ = 0.9841 and the lowest RMSE values. These models’ robustness was further validated by comparing the LOOCV results, which showed low prediction errors and good predictive coefficients (Q^2^ = 0.9777). Similarly, the EGA index provided reliable predictions for BP, E, MV, with *R* ^2^ values of 0.9207, 0.9269, and 0.9561, respectively, and strong LOOCV performance (Q^2^ = 0.9039, 0.9072, and 0.9486). The MECI index showed a reliable model for FP with *R* ^2^ = 0.8406 and Q^2^ = 0.8100. On the other hand, MP using the EABC index (*R* ^2^ = 0.2510, Q^2^ = 0.1464) and ST using the AECI index (*R* ^2^ = 0.1404, Q^2^ = 0.0299) showed poorer accuracy in prediction. The computed regression models are generally stable and not significantly over fitted, as shown by the close correlation between the regression and LOOCV results. In contrast, the comparatively poor predictive power for MP and ST shows that these properties could need more sophisticated nonlinear modelling techniques or additional molecular parameters to enhance prediction.

## 6 Conclusion

This study analyzed the efficacy of eccentricity-based topological indices in predicting the physicochemical properties of anti-cancer drugs within a QSPR framework. The regression models like linear, ridge, and elastic net were used to analyze the relationships between 13 eccentricity-based indices and a selected physicochemical property in hydrogen-depleted molecular graphs. The results indicated that several indices exhibited strong correlations with properties such as boiling point, molar refractivity, polarizability, and molar volume, thereby showing their ability to capture critical structural properties of molecular graphs. The ridge and elastic net, two regularized regression techniques, gave prediction results comparable to linear regression, suggesting the robustness and stability of the chosen eccentricity-based topological indices in univariate QSPR models. In general, the research has verified that eccentricity-based topological indices are computationally efficient and valuable for QSPR analysis of anti-cancer drugs. But, the present study is constrained by the relatively limited dataset. In order to enhance predictive performance and model generalization, future research may investigate the use of larger datasets and advanced machine learning techniques.

## Data Availability

The original contributions presented in the study are included in the article/supplementary material, further inquiries can be directed to the corresponding author.
